# Clinical and genetic findings in patients with congenital cataract and heart diseases

**DOI:** 10.1186/s13023-021-01873-7

**Published:** 2021-05-31

**Authors:** Xinru Li, Nuo Si, Zixun Song, Yaqiong Ren, Wei Xiao

**Affiliations:** 1 Department of Ophthalmology, Shengjing Hospital of China Medical University, Shenyang, 110004 Liaoning China; 2 Plastic Surgery Hospital, Chinese Academy of Medical Sciences and Peking Union Medical College, Beijing, China; 3 Institute of Basic Medical Sciences Chinese Academy of Medical Sciences, School of Basic Medicine Peking, Union Medical College, Beijing, China

**Keywords:** Congenital cataract, Congenital heart diseases, Microarray-based comparative genomic hybridisation, Whole exome sequencing, DECIPHER, Down’s syndrome, Atrial septal defect

## Abstract

**Background:**

Congenital cataract (CC) and congenital heart disease (CHD) are significant birth defects. In clinical practice, the concurrence of CC and CHD is frequently observed in patients. Additionally, some monogenic diseases, copy number variation (CNV) syndromes, and diseases associated with intrauterine infection involve both cataract and heart defects. However, little is known about the association between CC and CHD. Here, we characterised the demographic, clinical, and genetic features of patients with CC and heart defects.

**Methods:**

Medical records for 334 hospitalised patients diagnosed with CC were reviewed. Demographic and clinical features of patients with CC with and without CHD were compared. Clinical and genomic information for patients with ‘cataract’ and ‘cardiac defects’ were reviewed from Database of Chromosomal Imbalance and Phenotype in Humans using Ensembl Resources (DECIPHER). Microarray-based comparative genomic hybridisation and whole-exome sequencing were performed in 10 trio families with CC and CHD to detect de novo genomic alterations, including copy number variants and single nucleotide changes.

**Results:**

In a retrospective analysis of 334 patients with CC over the past 10 years at our hospital, we observed a high proportion of patients (41.13%) with CHD (including innocent CHD, which reported as left-to-right shunt in echocardiography test). The CC with CHD group had higher incidences of preterm birth and Down’s syndrome than the CC without CHD group. Atrial septal defect was the most frequent heart defect. A total of 44 cases with cataracts and heart diseases were retrieved from Database of Chromosomal Imbalance and Phenotype in Humans using Ensembl Resources (DECIPHER). In total, 52 genomic alterations were reported, 44% of which were de novo germline variants. In the 10 trio families with CC and CHD, we found de novo CNVs responsible for two well-known chromosomal disorders and identified a novel pathogenic mutation in *GJA8* responsible for CC.

**Conclusions:**

We observed significant associations between CHD and CC in our 10-year patient cohort. Based on the cohort and data from DECIPHER, developmental syndromes in some patients were due to genetic defects, thus explaining the concurrence of CC and CHD. Additionally, we detected de novo mutations as an independent cause of cataracts. Our findings suggest that developmental syndromes in patients with CC deserve more attention in clinical practice by ophthalmologists.

**Supplementary Information:**

The online version contains supplementary material available at 10.1186/s13023-021-01873-7.

## Background

Congenital cataract (CC) is one of the main causes of visual impairment and blindness in children. Opacity of the lens prevents light from entering the eye, thereby stunting the retinas and optic nerves and leading to amblyopia or blindness. About a quarter of CC cases are caused by genetic factors [1, 2], and other pathogenic mechanisms include infection and trauma [3]. Congenital heart disease (CHD) is one of the most common congenital malformations in humans [4]. The aetiology involves both environmental and genetic factors [5]. The lens and heart belong to different systems and develop from different germ layers; the lens develops from the ectoderm [3] and the heart develops from the mesoderm [6]. Despite these differences, some clinicians have made key observations suggesting that they may be linked. Among patients with CC who visit ophthalmology departments, a portion also show CHD [7, 8].

Furthermore, among patients with CHD who visit cardiology departments, a portion also show ocular abnormalities involving CC [9]. However, few studies have focused on describing and explaining these observations [7, 9]. Over a period of longer than 10 years at our hospital, one of the main hospitals for the diagnosis and treatment of CC in northeast China, we have observed that patients with CC have a higher incidence of CHD than that in the general population. Accordingly, echocardiography has been used as a routine preoperative test for CC in the last ten years.

Some aetiological bases, including genetic mutations, underlying CC and CHD have been revealed respectively. More than 100 genes and nearly 200 loci have been identified in monogenic forms of CC [3]. Copy number variation (CNV), leading to the deletion or duplication of functional DNA fragments, is one of the main genetic mechanisms underlying CHD. In addition, some monogenic diseases, CNV syndromes, and intrauterine infection-related diseases involve concurrent cataract and heart defects, such as oculo-facio-cardio-dental syndrome (OFCD) caused by *BCOR* mutations [10], *FBXL4*-related encephalomyopathic mitochondrial DNA depletion syndrome by *FBXL4* mutations [11], Sengers syndrome caused by *AGK* mutations [12], Nance-Horan syndrome caused by *NHS* mutations [13], Down’s syndrome caused by trisomy 21 [14], and congenital rubella syndrome caused by rubella virus intrauterine infection [15]. The frequent co-occurrence of CC and heart defects prompted us to speculate whether the two defects share a common aetiological basis.

In the present study, we characterised the demographic and clinical features of patients with CC and heart defects by reviewing the 10-year patient cohort at our hospital. We also attempted to identify the individual and/or common causes underlying these two defects based on genetic findings for reported patients and newly recruited trio-families.

## Results

### Statistical analysis of clinical data

Based on early observations that CHD is frequently associated with CC, echocardiography for evaluating heart defects has become a routine preoperative examination for patients with CC in the past 10 years. In total, 334 patients with CC referred to our hospital in 2010–2020, including 248 who underwent echocardiography examination. We found that 41.13% of patients with CC (102/248) also had CHD. For the reason that left-to-right shunt in echocardiography also represent innocent CHD, such as small ASD (atrial septal defect)or PFO (patent foramen ovale), the observed CHD in this study include major heart structural defect and innocent CHD which cannot be classified as either physiologic or pathologic [16–22]. According to echocardiography results, these 248 patients were divided into two groups: CC with CHD (N = 102) and CC without CHD (N = 146). Medical records for these patients were evaluated, and the two groups were compared with respect to general information, family history, and clinical manifestations (Table 1). There were no significant differences in the proportions of males and females between the two groups. With respect to obstetric history, the proportion of premature births was significantly higher in the CC with CHD group (15.69%) than in the CC without CHD group (4.11%) (*P* = 0.002). Additionally, 2.94% of patients in the CC with CHD group had pregnancy with rubella, diabetes, rash, or cold, which were not reported in the CC without CHD group; however, there were no statistically significant differences in these conditions. Cataracts could be inherited from parents or occur de novo. There was no difference in the family history of CC between CC with CHD and without CHD groups. However, cataracts typically occurred de novo in both groups, with frequencies of 95.1% (N = 97) and 90.41% (N = 132) in CC with CHD and without CHD, respectively. For patients who had affected parents, maternal inheritance was more common. As for ophthalmic manifestations, most patients had bilateral cataracts and presented pure lens opacity, and there was no difference between the two groups. We also observed other ophthalmic manifestations in both groups, such as vitreous opacity, strabismus, and nystagmus; however, the frequencies of these phenotypes did not differ between the two groups. Developmental abnormalities were found in some patients, including brain CT abnormalities, diagnosed Down’s syndrome, developmental retardation, deafness, limb malformation, mental retardation, external auditory canal atresia, and anal atresia. It is noteworthy that four patients were diagnosed with Down’s syndrome in the CC with CHD group, and the frequency differed between the two groups (*P* = 0.028). Some other extraocular and extracardiac diseases/symptoms were occasionally identified in each group, but the differences in frequencies between groups were not significant.

**Table 1:** Clinical manifestations in patients with congenital cataracts with and without congenital heart defects

Variable	CC with CHD (N)	CC without CHD (N)	*P*-value
Total number of patients	102	146	
Gender			0.856
Male	54	79	
Female	48	67	
Obstetric history			
Premature birth	16	6	0.002
Pregnancy with rubella, diabetes, rash, and cold	3	0	0.068
Family history of cataract			0.601
Maternal inheritance	3	8	0.533
Paternal inheritance	0	2	0.541
De novo	97	132	0.172
Unavailable data	2	4	1
Cataract			0.897
Unilateral	28	39	
Bilateral	74	107	
Ocular findings			
Pure lens opacity	70	93	0.421
Vitreous opacity	12	9	0.119
Strabismus	8	21	0.115
Nystagmus	8	13	0.768
Ocular dysplasia	4	3	0.45
Persistent hyperplastic primary vitreous (PHPV)	4	3	0.45
Lens dislocation	3	6	0.74
Angular nearly	1	0	0.411
Epicanthus	1	0	0.411
Fluid area above right eye bulb	1	0	0.411
Congenital glaucoma	1	3	0.646
Hypermyopia	0	1	1
Hyperpresbyopia	0	1	1
Congenital pupil atresia	0	1	1
Ptosis	0	1	1
Posterior cystic mass of the eyeball	0	1	1
Developmental abnormalities			
Brain CT abnormalities	11	12	0.493
Down's syndrome	4	0	0.028
Developmental retardation	3	0	0.068
Deafness	3	2	0.405
Limb malformation	2	0	0.168
Mental retardation	1	2	1
External auditory canal atresia	0	1	1
Anal atresia	0	1	1
Other diseases			
Benign Myospasm	1	0	0.411
Colopathy	1	1	1
Leg cramps	1	0	0.411
Hypothyroidism	1	0	0.411
Epilepsy	1	1	1
Congenital diabetes	0	1	1
Galactosemia	0	1	1
Congenital heart defects			
Atrial Septal Defect (ASD)*	73	0	0
Patent ductus arteriosus (PDA)	17	0	0
Ventricular septal defect (VSD)	15	0	0
Tricuspid regurgitation, Ebstein anomaly, tricuspid insufficiency	7	0	0.002
Pulmonary hypertension (PAH)	6	0	0.004
Pulmonary valve stenosis	5	0	0.011
Permanent left superior lumen artery	3	0	0.068
Pulmonary stenosis	2	0	0.168
Mitral regurgitation	1	0	0.411
Left ventricular diastolic function decreased	1	0	0.411
Left bundle branch block	1	0	0.411
Coronary artery aortic fistula	1	0	0.411
Fallot's triad	1	0	0.411

CC: Congenital cataract

CHD: Congenital heart defects

^*^ASD defined as all symptoms with a left to right atrial shunt

**Fig. 1:**
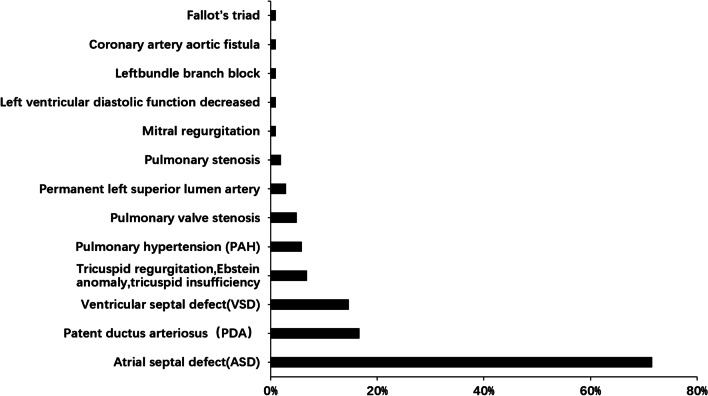
Types of heart defects in patients with congenital cataracts in our 10 years cohort study

When focusing on cardiac phenotypes in the CC with CHD group, atrial septal defect (ASD) (73, 71.57%) (We defined ASD as all symptoms with a left-to-right atrial shunt.) was the most common type of heart disease, followed by patent ductus arteriosus (17, 16.67%) and ventricular septal defect (VSD) (15, 14.7%). Most patients had a single type of heart defect (79, 77.5%), and a few patients had complex heart defects (23, 22.5%) (Fig. 1).

### Review of patients with cataract and heart disease in DECIPHER

**Fig. 2:**
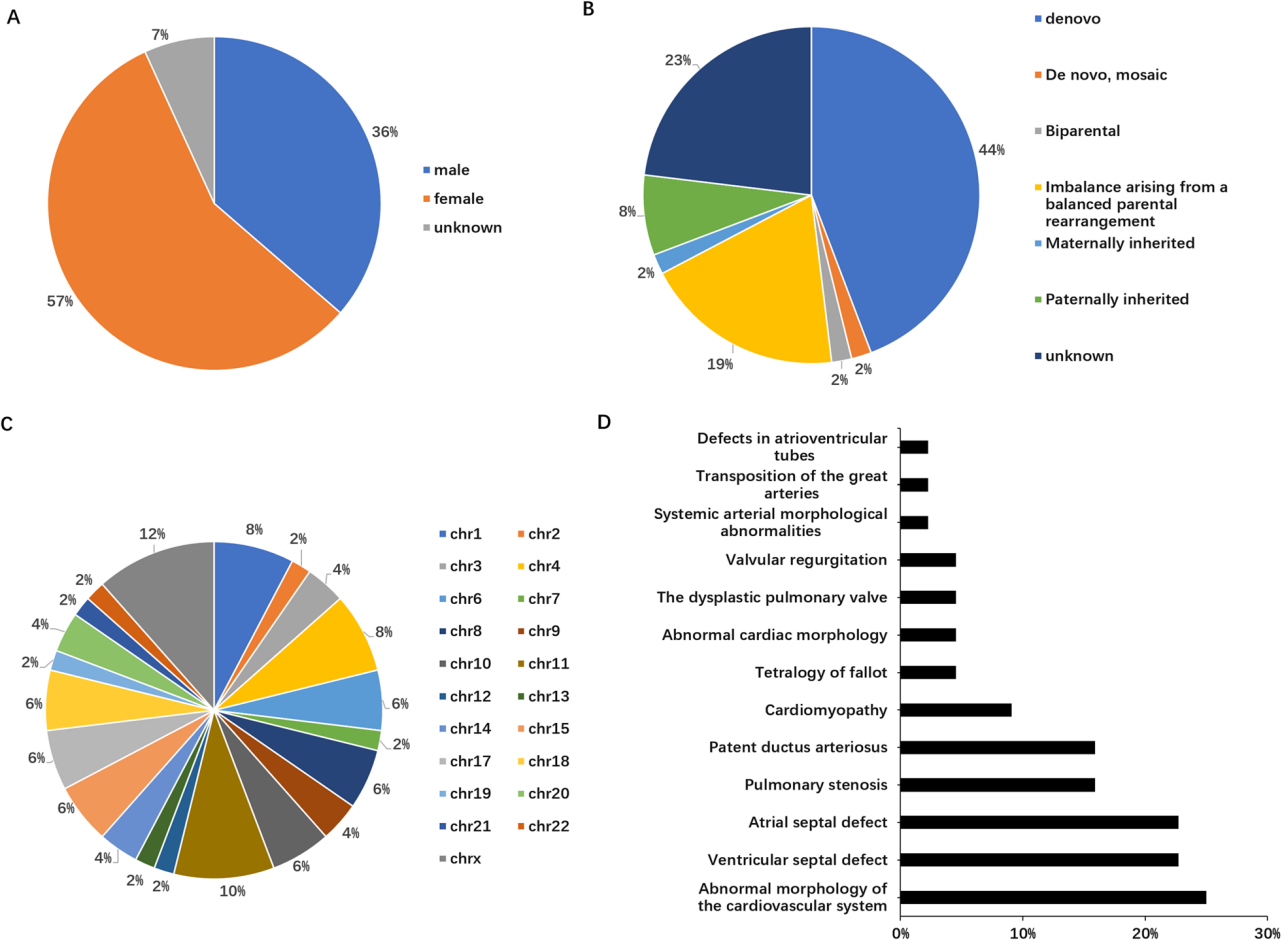
Demographic and genetic characters of patients with CC and CHD in DECIPHER. **A** Gender composition of 44 patients. **B** Types of variants detected in patients. **C** Chromosome distribution of variants. **D** Types of cardiac defects in patients

A total of 44 cases with cataract and heart diseases were retrieved from the Database of Chromosomal Imbalance and Phenotype in Humans using Ensembl Resources (DECIPHER) (Additional file 2: Table 1), of which 57% were female, 36% were male, and 7% had no reported gender. In total, 52 genomic alterations were reported in these patients. In terms of the origin of the variants, 44% were de novo germline variants, 19% were imbalanced arising from a balanced parental rearrangement, 8% were paternally inherited, 2% were biparental, 2% were maternally inherited, 2% were de novo mosaic variants, and 23% were of unknown inheritance. Except for chr5 and chr16, variants were found on each chromosome, with 12% on chrX, 8% on chr1, and 10% on chr11. VSD (N = 10, 22.7%) and ASD (N = 10, 22.7%) were the most frequent heart defects. Eleven patients with abnormal cardiovascular systems were reported without a specific type, and other heart defects included pulmonary stenosis, patent ductus arteriosus, cardiomyopathy, tetralogy of Fallot, dysplastic pulmonary valve, valvular regurgitation, systemic arterial morphological, transposition of the great arteries, and defects in the atrioventricular tube (Fig. 2).

### Genomic findings in family-trios with congenital cataract and congenital heart disease

**Fig. 3:**
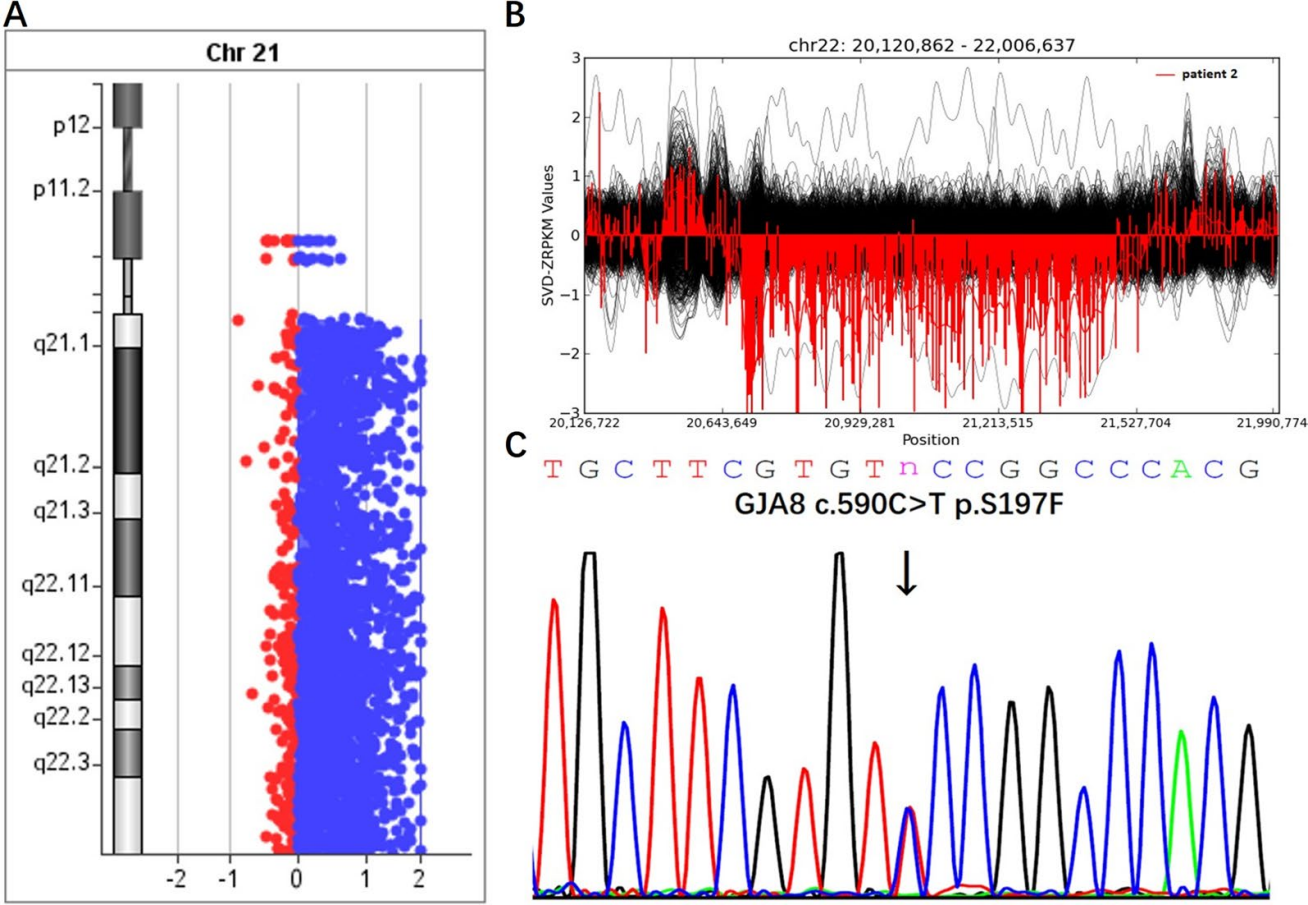
Pathogenic genomic alterations detected in family-trios with CC and CHD. **A** 21q11.2-q22.3 duplication detected using aCGH in patient 1. **B** 22q11.21 deletion detected by WES in patient 2. **C** *GJA8* missense mutation in patient 3

We recruited 10 family-trios with CC and CHD and performed genome-scale screening for genomic alterations by microarray-based comparative genomic hybridisation (aCGH) and/or whole-exome sequencing (WES). Since the patients were born to healthy parents and no pregnancy complications were reported, we focused on de novo genomic alterations that were large in size or had potential deleterious effects. These alterations were only detected in patients but not in their unaffected parents. De novo CNVs larger than 100 kb and de novo SNPs or insertions/deletions (InDels) interpreted as pathogenic or likely pathogenic are listed in Table 2 and Additional file 3: Table 2. De novo CNVs larger than 100 kb were detected in seven family trios. Among them, a 21q11.2-q22.3 duplication in patient 1 and 22q11.21 deletion in patient 2 are associated with specific diseases, Down syndrome and 22q11.2 deletion syndrome, respectively (Fig. 3A, B). Other de novo CNVs do not have known associations with diseases; nonetheless, they are absent in the DGV database documenting copy number polymorphisms in healthy individuals (Additional file 1: Fig. 1A–E). Pathogenic or likely pathogenic de novo SNPs in six genes were detected in five trio families. All of these SNPs were heterozygous in the patient and absent from the genomes of the parents. *GJA8* is a common pathogenic gene in CC. We detected a novel *GJA8* missense mutation (c.590C > T p.S197F) in patient 3 (Fig. 3C). In silico prediction using VarCards (http://varcards.biols.ac.cn/) indicated that it was deleterious, with a D: A algorithm score of 22:23 and a damaging score of 0.96, and the mutation was extremely rare in public datasets, with no reported allele frequency. Mutations in *POLG* are responsible for progressive external ophthalmoplegia, which includes cataracts as an additional symptom. However, patient 8 with a *POLG* mutation did not present weakness of the external eye muscles or exercise intolerance. Biallelic mutations of *YIF1B* could lead to an autosomal recessive neurodevelopmental disorder, Kara-Barakat-Masson syndrome (KABAMAS), characterized by global developmental delay, motor delay, ocular abnormalities, and nervous system change [23, 24]. The visual deficits in KABAMAS included strabismus, optic atrophy, eye to eye contact, nystagmus. However, a cataract was not reported in KABAMAS patients [24]. Patient 10 carrying a heterozygous mutation of *YIF1B,* and present no typical KABAMAS clinical manifestations. Other genes with detected pathogenic or likely pathogenic mutations were either not associated with any diseases (*PRAMEF12*, *MDN1*) or associated with unrelated phenotypes (*LIPC*) (Additional file 1:Fig. 1F–J).

**Table 2:** De novo genomic changes in trio families with congenital cataract and congenital heart diseases

Patient	Gender	Age	Ophthalmic Feature	Cardiac Feature		De novo CNVs*			De novo SNPs/Indels#	
					Changes		Length(kb)	Diseases	Changes	Disease
P1	Male	6 years	Left eye developmental membranous cataract, bilateral vitreous opacity	Ventricular septal defect	21q11.2-q22.3 dup		146,608.368	Down's syndrome	ND	NA
P2	Male	3 months	Bilateral congenital nuclear cataract	Atrial septal defects	22q11.21 del		685.775	22q deletion syndrome	ND	NA
P3	Female	2 months	Bilateral congenital cataract	Atrial septal defect, coronary sinus dilation, persistent left superior vena cava (PLSVC)	ND		ND	NA	GJA8 c.590C > T p.S197F	Cataract
P4	Female	6 months	Bilateral congenital coralliform cataract	Atrial septal defects	10q11.21 del		125.435	NA	ND	NA
P5	Female	5 years	Bilateral developmental pulverulent cataract	Atrial septal defects	17q21.2 dup		201.614	NA	PRAMEF12 c.1372C > G p.R458G	NA
									LIPC c.317C > T p.A106V	Hepatic lipase deficiency (AR)
P6	Female	4 years	Bilateral congenital total cataract	Atrial septal defects	13q12.13 dup		197.14	NA	ND	NA
P7	Male	5 months	bilateral Congenital total cataract	Left myocardial hypertrophy, atrial septal defects	7q22.3 del		339.764	NA	ND	NA
P8	Male	2 months	Bilateral congenital cataract	Atrial septal defects	4q34.3-q35.1 dup		2000.726	NA	POLG c.2669A > C p.D890A	Mitochondrial DNA depletion syndrome, progressive external ophthalmoplegia
P9	Female	1 year and 3 months	Bilateral perinuclear congenital cataract	Atrial septal defects	ND		ND	NA	MDN1 c.16697A > G p.Y5566C	NA
P10	Female	2 year and 3 months	Left eye congenital cataract	Atrial septal defects	ND		ND	NA	YIF1B NM_001039672 c.709G > A p.V237I	Kaya-Barakat-Masson syndrome, (AR)

ND: not detected; NA: not available; CNV: copy number variation; SNP: single nucleotide polymorphism; AR: autosomal recessive inheritance

^*^ > 100 kb

^#^ Likely pathogenic or pathogenic according to the ACMG/AMP 2015 guideline

Atrial septal defects defined as all symptoms with a left to right atrial shunt

## Discussion

Over 20 years of experience in ophthalmology has revealed that CC is frequently associated with CHD. However, barely any study was performed to describe and explain this observation [7, 9]. As one of the hospitals receiving and treating most patients with CC in northeast China, we have also observed this interesting association in our patient cohort and have used echocardiography as a routine preoperative examination for patients with CC for over 10 years. In a retrospective study of 334 patients with CC in the past 10 years at our hospital, we found a high frequency (41.13%) of CHD (including innocent CHD, which has left-to-right blood shunt). This was significantly greater than the 1% incidence of CHD in the general population worldwide [5, 25] and reinforces the significant association between CHD and CC [7, 9]. Further, patients with CC with and without CHD were compared with respect to gender, obstetric history, family history, and ocular, cardiac, and systematic manifestations. We found that the CC with CHD group had higher incidences of preterm birth and Down’s syndrome than those in the CC without CHD group. The risk of preterm birth may be associated with CHD or related to other maternal genetic factors or viral infection affecting embryogenesis [26]. We also observed that Down’s syndrome is significantly more frequent in the CHD group than in the non-CHD group. These results are consistent with those of the previous report indicating a possible relationship between CC and CHD in patients with Down’s syndrome [27].

CC with CHD is often reported in rare syndromes with well-defined genetic or chromosomal mutations or in syndromes resulting from intrauterine infection, such as OFCD syndrome caused by *BCOR* mutations [10], recurrent rearrangements of chromosome 1q21.1 [28], Down's syndrome [14], and congenital rubella syndrome [15]. *BCOR* acts as a pivotal multisystem transcriptional regulator during early embryogenesis. Recurrent rearrangements of chromosome 1q21.1 and Down's syndrome are both caused by chromosomal mutations involving many different genes, and congenital rubella syndrome is caused by multisystem interference by infection in early development. Accordingly, we reasoned that early in embryonic development, the eye and heart might be regulated by a set of reciprocally regulated genes, genes that function independently in these organs might be located in close proximity on the chromosome, or the foetus might be susceptible to infection.

We also reviewed genomic findings for 44 cases with CC and CHD in DECIPHER. We found no significant difference in sex ratio, the type, and proportion of heart disease, or clinical features between these cases and our patient cohort. The main types of heart disease were ASD and VSD. Genomic changes, including structural and nucleotide changes, were distributed across different chromosomes, showing no obvious enrichment (Additional file 2: Table 1). Nonetheless, we found pathogenic mutations for specific developmental syndromes with CC and CHD as manifestations, including *BCOR* mutations for OFCD syndrome (DECIPHER ID 262217, 267974, and 303226), and the 11q21.1 deletion for 1q21.1 rearrangements of chromosome 1q21.1 (DECIPHER ID 323303).

We further performed a comprehensive genetic analysis of 10 trio families with CC and CHD to determine the underlying genetic causes. These patients were born to non-consanguineous healthy parents. Therefore, de novo genomic changes are more likely to be the cause of the phenotype, if other risk factors can be excluded, such as viral infection. Consistent with the results of the DECIPHER analysis, we found de novo CNVs responsible for two well-known chromosomal disorders. Patient 1 was a 6-year-old boy with a developmental membranous cataract in the left eye, bilateral vitreous opacity, and VSD. By array CGH, we detected a de novo 21q duplication that could lead to Down’s syndrome. The patient presented obvious eye spacing and mental retardation. His parents indicated that the patient had been diagnosed with Down’s syndrome; however, no medical records were provided. About half of patients with Down’s syndrome are born with CHD. Ophthalmological abnormalities, including CC, are frequently found in patients with Down syndrome [29]. 21q11.2-q22.3 duplication detected in patient 1 overlaps with the Down syndrome critical region on chromosome 21 which contains the major genes responsible for the disease's pathogenesis [30]. Patient 2 was a 3-month-old male with bilateral congenital nuclear cataract and ASD. He was born prematurely. A genetic analysis showed that he carried a de novo deletion on 22q11. The deletion overlapped with pathogenic deletions causing 22q11 deletion syndrome, which usually includes multiple organ dysfunctions, such as abnormalities of the heart, palatal abnormalities, thyroid hypofunction, facial deformity, and mental diseases [31, 32]. Some cases have ocular symptoms, such as strabismus, ametropia, ocular dysplasia, and cataract [33]. 22q11 deletion syndrome is classified as proximal, central, and distal deletions [34]. The 22q11.21 deletion detected in patient 2 was a central deletion, and it does not contain the famous development gene *TBX1*. Nonetheless, genes functionally related to heart and eye development were found in the central deleted region. The region contains the *CRKL* gene, which plays an important role in the development of the heart [35, 36]. The *Crkl* gene is related to lens formation in mice [37]. *ZNF74* is found at a relatively high frequency in patients with CHD with deletions/amplifications in the 22q11.2 region [38]. *MED15* is related to heart development [39]. A *SCARF2* microdeletion has been found in a patient with abnormal eye development by Migliavacca et al. [40]. The co-existence of defects in different tissues or organs could result from the dysregulation of different genes within the same rearranged chromosomal region. The identification of de novo pathogenic CNVs in our patients suggests that the co-occurrence of CC and CHD could be explained by a shared underlying chromosomal disorder. Thus, in clinical practice, both ophthalmologists and cardiologists should pay more attention to chromosomal disorders when patients present both CC and CHD.

We also identified a novel pathogenic mutation in the *GJA8* gene responsible for CC in patient 3. The patient was a 2-month-old female with bilateral CC, ASD, coronary sinus dilation, and persistent left superior vena cava. She carried a deleterious de novo *GJA8* missense mutation (c. 590C > T p. S197F). *GJA8* encodes a gap junction protein, which is crucial for maintaining ionic and water balance and for the transparency and optical properties of the lens [41]. It is a well-known causative gene for cataracts. No evidence supported the causal relationship between *GJA8* and heart defects, although other gap junction protein-coding genes, *GJA1* and *GJA5*, play crucial roles in heart development and function [42–44]. It is likely that CC and CHD in patient 3 are two independent phenotypes with different underlying causes. The heart defects could have resulted from distinct genetic, environmental, and stochastic factors.

## Conclusions

In this study, we observed significant associations between CHD and CCs in our 10-year patient cohort. Detailed clinical and genomic findings for both the patient cohort and cases in DECIPHER were evaluated. We found some patients had developmental syndromes due to genetic defects, especially CNVs affecting contiguous or reciprocally regulated genes involved in eye and heart development. Our findings suggest that developmental syndromes in patients with CC are underestimated in clinical practice by ophthalmologists. We also detected several de novo mutations in trios; however, we could not determine whether the de novo mutations detected in our trios are common or independent causes of heart and ocular anomalies. Comprehensive genetic analyses based on larger sample sizes and functional studies could provide further insight into the association between CC and CHD.

## Methods

### Subjects

The medical records for 334 hospitalised patients diagnosed with CC in the Department of Ophthalmology of Shengjing Hospital, Shenyang, China, from 2010 to 2020 were reviewed. Gender, age, family history, past medical history, birth status, ocular and systemic physical examination, and imaging data were collected. In total, 248 patients completed echocardiography and were included in statistical analyses and the collation of medical records. In addition, 10 trio families we recruited in 2018–2019. In these trio families, patients with CC and CHD were born to their healthy parents and no other family history or pregnancy complications were reported. Peripheral blood from patients and parents was collected for genetic analysis after obtaining informed consent. The study was approved by the institutional review board of China Medical University.

### Statistical analysis of clinical data

Clinical data for 248 patients who underwent echocardiography were reviewed. General information, including date of birth, gender, and family history were collected. Cataracts in one or both eyes and other ocular abnormalities were identified based on the two-dimensional or three-dimensional ocular ultrasound and ophthalmic examinations. CHD was diagnosed by echocardiography and relevant systemic examinations. All 248 patients were divided into two groups, CC with or without CHD. Categorical variables were compared between the two groups by a chi-squared test or Fisher's exact test, and values of *P* < 0.05 were considered statistically significant. Statistical analyses were performed using IBM® SPSS® Statistics version 24 (IBM Corp., Armonk, NY, USA).

### Review of patients with CC and CHD in DECIPHER

Reported clinical and genomic information for patients with ‘cataract’ and ‘cardiac defects’ were reviewed from the Database of Chromosomal Imbalance and Phenotype in Humans using Ensembl Resources (DECIPHER). The term ‘cataract’ was used for the search and patients with cardiac defects were manually selected according to the documented phenotypes. Clinical information (gender, inheritance, and type of heart disease) and genomic findings (genes with mutations and altered genomic regions) for 44 patients were reviewed and analysed.

### Genetic testing for de novo genomic alterations

De novo genomic alterations were detected in 10 CC with CHD trios at a genome-wide scale. CNVs were detected by microarray-based comparative genomic hybridisation (aCGH), and whole-exome sequencing (WES) was used to detect SNPs and InDels. When DNA was not sufficient to detect CNVs using aCGH, WES results were used to detect CNVs using the CoNIFER tool based on read depth [45]. Genetic testing was performed for each patient and his/her unaffected parents, and the status of genomic alterations (i.e. de novo or not) were determined accordingly. Eight trios were evaluated using aCGH and WES. Two trios were only evaluated by WES owing to insufficient DNA. For aCGH assays, the 4 × 180 K SurePrint G3 Human CGH Microarray was used (Agilent. Santa Clara, CA, USA), and Feature Extraction and Agilent Genomic Workbench were used for analyses. The Database of Genomic Variants (DGV) (http://dgv.tcag.ca) and DECIPHER (https://decipher.sanger.ac.uk/) were used to determine the pathogenicity of candidate CNVs as follows. (1) A full CNV region reported in the general population in DGV n ≥ 3 times or a CNV region without genes was defined as a benign CNV. (2) A full CNV region reported in the general population in DGV n < 3 times and including genes overlapping with CNV syndromes reported in DECIPHER or reported as causes of a similar phenotype in PubMed and OMIM was defined as a pathogenic CNV. (3) The remaining were defined as uncertain pathogenic [46–48]. For WES assays, pathogenic de novo variants were interpreted according to the ACMG/AMP 2015 guideline and VarCards (http://varcards.biols.ac.cn/). Sanger sequencings were used to verify candidate SNPs/InDels.

## Supplementary Information

**Additional file 1: Figure 1**. aCGH results for CNVs of uncertain pathogenicity using the Agilent Cytogenomics tool and Sanger sequencing results for other pathogenic/likely pathogenic de novo SNPs. A–E show the 10q11.21 deletion in patient 4, 17q21.2 duplication in patient 5, 13q12.13 duplication in patient 6, 7q22.3 deletion in patient 7, 4q34.3-q35.1 duplication in patient 8, in order. F–J represent Sanger sequence results for mutations in PRAMEF12 (c.1372C>G p.R458G) and LIPC (c.317C>T p.A106V) in patient 5, POLG (c.2669A>C p.D890A) in patient 8, *MDN1* (c.16697A>G p.Y5566C) in patient 9, and *YIF1B* (NM_001039672 c.709G>A p.V237I) in patient 10

**Additional file 2: Table 1**. Genomic findings in patients with cataract and heart diseases in DECIPHER database

**Additional file 3: Table 2**. Genomic location of CNVs in patients with congenital cataract and congenital heart diseases in trio families

## Data Availability

The datasets used and/or analysed in the current study are available from the corresponding author on reasonable request.

## Abbreviations

CC: Congenital cataract
CHD: Congenital heart disease
CNV: Copy number variation
DECIPHER: Database of chromosomal imbalance and phenotype in humans using ensembl resources
aCGH: Microarray-based comparative genomic hybridisation
WES: Whole exome sequencing
DGV: Database of genomic variants
OFCD: Oculo-facio-cardio-dental syndrome
SNP: Single nucleotide polymorphism
InDel: Insertion–deletion
OMIM: Online Mendelian inheritance in man database
ACMG/AMP: American college of medical genetics and genomics and the association for molecular pathology
ASD: Atrial septal defect
VSD: Ventricular septal defect
