# Supplementary figures and images for: Clinical and genetic findings in patients with congenital cataract and heart diseases
Source: Orphanet J Rare Dis. 2021 May 31;16:242. doi: 10.1186/s13023-021-01873-7 (PMC8165991; doi:10.1186/s13023-021-01873-7)

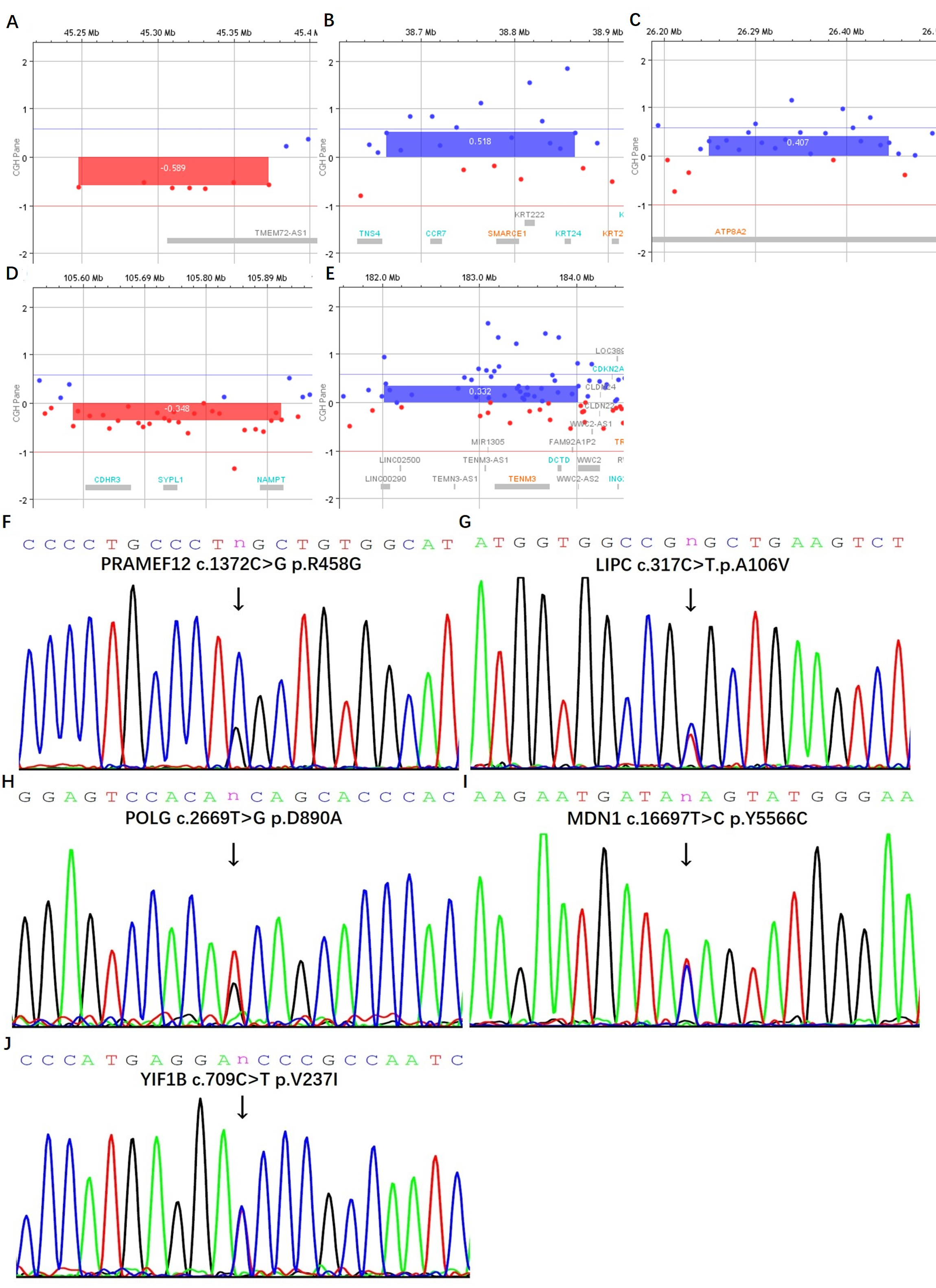

Supplement: Supplementary file 1 — Additional file 1: Figure 1. aCGH results for CNVs of uncertain pathogenicity using the Agilent Cytogenomics tool and Sanger sequencing results for other pathogenic/likely pathogenic de novo SNPs. A–E show the 10q11.21 deletion in patient 4, 17q21.2 duplication in patient 5, 13q12.13 duplication in patient 6, 7q22.3 deletion in patient 7, 4q34.3-q35.1 duplication in patient 8, in order. F–J represent Sanger sequence results for mutations in PRAMEF12 (c.1372C>G p.R458G) and LIPC (c.317C>T p.A106V) in patient 5, POLG (c.2669A>C p.D890A) in patient 8, MDN1 (c.16697A>G p.Y5566C) in patient 9, and YIF1B (NM_001039672 c.709G>A p.V237I) in patient 10 [file 13023_2021_1873_MOESM1_ESM.png]
