# Supplementary material for: Clinical and genetic findings in patients with congenital cataract and heart diseases
Source: Orphanet J Rare Dis. 2021 May 31;16:242. doi: 10.1186/s13023-021-01873-7 (PMC8165991; doi:10.1186/s13023-021-01873-7)
Supplement: Supplementary file 3 — Additional file 3: Table 2. Genomic location of CNVs in patients with congenital cataract and congenital heart diseases in trio families [file 13023_2021_1873_MOESM3_ESM.docx]

| **Supplementary table 2.Genomic location of CNVs** | | |
| --- | --- | --- |
| **patient** | **de novo CNVs changes** | **CNV Genomiccoordinates (GRCh37/hg19)** |
| patient 1 | 21q11.2 - q22.3dup | 1481949-148090317 |
| patient 2 | 22q11.21del | 20720862-21406637 |
| patient 3 | ND | ND |
| patient 4 | chr10q11.21del | 45247685-45373120 |
| patient 5 | chr17q21.2dup | 38663148-38864762 |
| patient 6 | chr13q12.13dup | 26248721-26445861 |
| patient 7 | chr7q22.3del | 105583993-105923757 |
| patient 8 | 4q34.3 - q35.1dup | 182017702-184018428 |
| patient 9 | ND | ND |
| patient 10 | ND | ND |
|  |  |  |
| ND: not detected | |  |
| del: deletion |  |  |
| dup: duplication | |  |
